# Techno-economic data for a multi-model approach to decarbonisation of the Irish private car sector

**DOI:** 10.1016/j.dib.2017.10.006

**Published:** 2017-10-07

**Authors:** Eamonn Mulholland, Fionn Rogan, Brian P.Ó Gallachóir

**Affiliations:** a Energy Policy and Modelling Group, MaREI Centre, Environmental Research Institute, University College Cork, Cork, Ireland,; b School of Engineering, University College Cork, Cork, Ireland,

## Abstract

These data and analyses support the research article “From technology pathways to policy roadmaps to enabling measures – A multi-model approach” Mulholland et al. (2017) [1]. This article uses 3 models – an optimization model of the Irish energy system (Irish TIMES), a simulation model of the Irish private transport sector (CarSTOCK), and a market share algorithm used to provide a behavior rich representation into the multi-modelling process. Each of these models are linked to provide a technology pathway, policy roadmap, and finally identify the enabling measures of the private transport sector in a low-carbon Ireland moving toward 2050. The article is organized in the same order, firstly providing the key modelling assumptions and operability of Irish TIMES, secondly for CarSTOCK, and finally for the market share algorithm. All data is supplied within this article.

**Specifications Table**

**Table:** 

Subject area	*Energy Modelling*
More specific subject area	*Multi-modelling approach of the private transport sector*
Type of data	*Text-file fitted with supplementary graphs and tables*
How data was acquired	*Irish TIMES data was acquired from the Pan European TIMES (PET) model, the Economic and Social Research Institution (ESRI), and a wide ranging body of literature reviews.*
	*CarSTOCK data was acquired from the Sustainable Energy Authority of Ireland (SEAI), ESRI, the National Car Test (NCT), the Vehicle Registration Unit (VRU), and a range of data taken from literature reviews.*
	*Data for the Market Share Algorithm was populated with data from the Irish TIMES model, and the CarSTOCK model.*
Data format	*Raw model input data*
Experimental factors	*N/A*
Experimental features	*N/A*
Data source location	*PET data related to the EU27, Iceland, Switzerland, Norway, and Balkan Countries. The remainder of the data was made specific to Irish TIMES.*
Data accessibility	*All data is provided within this article*

**Value of the data**

• This data provides transparency behind the modelling assumptions and methodology used for a multi-modelling approach used to decarbonize the private transport energy sector in Ireland.
• While the data is Ireland specific, it serves as a guideline for the scientific community to ways to replicate similar modelling methods designed for other regions at a local, national, or international level.
• It provides valuable insights into the sources available at a national level which most European member states have freely available, and which can be used to replicate the modelling methods provided in the accompanying Energy article.

## 1 Data

The dataset within this article provides information on the cost, fuel economy and mileages for the private car sector in Ireland (Table 1, Table 7, and Table 10). Furthermore, data pertaining to the key assumptions used by the Irish TIMES linear optimisation model are included, such as wind energy capacities (Table 4), Irish biofuel energy potential (Table 5), Irish biofuel costs (Table 6).

**Table 1:** Private car technologies and associated investment costs.

Technology	Description	Investment Cost – 2010 (k€/vehicle)	Investment Cost – 2050 (k€/vehicle)
TCARSBDL101	Biodiesel Car	9.8	10.7
TCARSDME110	DME Car	11.5	10.5
TCARSDST101	Diesel Car	8.5	8.5
TCARSDST210	Hybrid Diesel Car	13.4	12.4
TCARSELC110	Electric Car	20.7	10.0
TCARSETH101	Ethanol Car	9.0	9.9
TCARSFTD110	FT-Diesel Car	9.7	10.7
TCARSGAS101	CNG Car	9.8	10.7
TCARSGH2110	Internal Combustion Hydrogen Car (Compressed)	13.5	12.5
TCARSGH2210	Fuel Cell Hydrogen Car (Compressed)	14.0	13.0
TCARSGSL101	Gasoline Car	8.6	9.5
TCARSGSL201	Hybrid Gasoline Car	16.5	11.2
TCARSLH2110	Internal Combustion Hydrogen Car (Liquefied)	13.5	12.5
TCARSLPG101	LPG Car	9.6	10.5
TCARSMtaH101	IC Methanol Car	9.0	9.9
TCARSMtaH210	Fuel Cell Methanol Car	14.0	13.0
TCar_PIH	Plug-in Hybrid Car	17.0	13.0

**Fig. 1:**
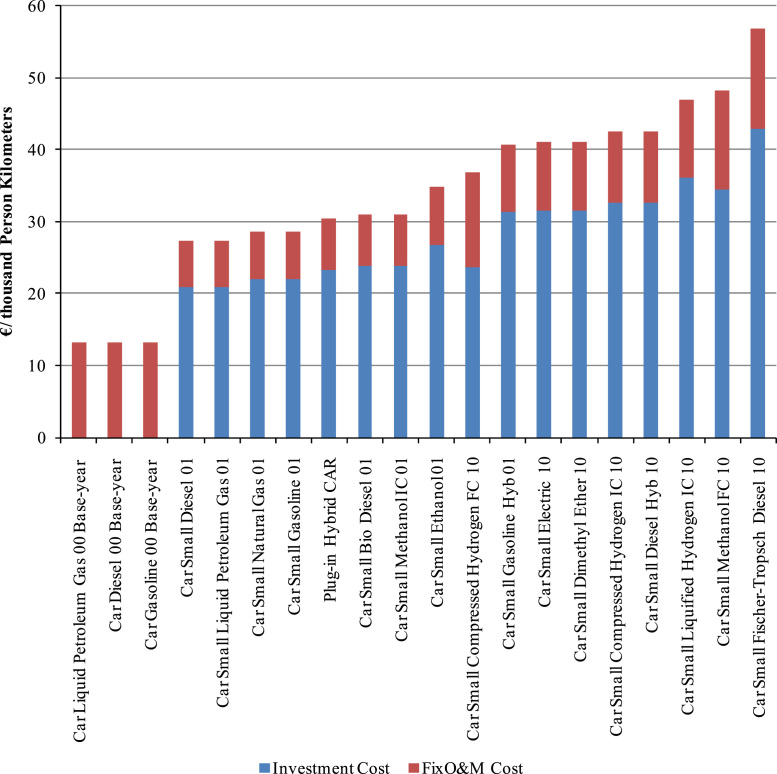
Comparison of private car investment and O&M costs.

Each technology has an associated investment cost as well as operational and maintenance costs. Fuel costs are also accounted for but these are endogenous to the model and are not classed as inputs. The technology costs can be arranged in order of increasing cost to give a cost curve of various technology options which can help identify which technologies may be chosen before others in the TIMES model. A sample of such a cost curve for select technologies (in this case a subset of the private car technologies) is given in Fig. 1 below. Other inputs for each technology include fuel type and efficiency.

## 2 Experimental design, materials and methods

### 2.1 Irish TIMES model operation and input assumptions

The Irish TIMES model is a linear optimisation model with an objective function to minimise total system cost (maximizes the total discounted surplus) subject to imposed constraints. Mathematical equations describe the relationships and interaction between the many technologies, drivers and commodities in Irish TIMES. While it is tempting to think of Irish TIMES as a simple ‘merit type’ model that chooses technologies simply from the least expensive to the most expensive to meet certain demands this is an oversimplification that leads to an incorrect understanding of the model value and dynamics. The richness of the Irish TIMES model is that it optimises across all sectors of the energy system for the full horizon and thus captures the interaction between sectors. The model simultaneously solves for the least cost solution subject to emission constraints, resource potentials, technology costs, technology activity and capability to meet individual energy service demands. In this way Irish TIMES allows technologies to compete both horizontally across different energy sectors and vertically through the time horizon of the model.

There are a large number of exogenous inputs to the Irish TIMES model. Many of these are characterizations of technology or commodity entities. There are also a number of endogenous inputs that are calculated by Irish TIMES and which are used in the final calculations for the model outputs. Some of relevant model inputs are presented in the following sections. This document serves as an overall review of these data with a further specific focus behind the private transport sector.

### 2.2 Technologies

In the Irish TIMES model, there are more than 1350 technologies for the supply-side and demand-side sectors of the economy. Each of these technologies has detailed technical parameters that can be changed and set by the user; some of these parameters include technology efficiency (e.g. heat rates, learning curves), technology lifetime, emission factors (CO2 and non-CO2) and availability. The data sources for most of these technologies are the IEA databases that were used to build the reference energy system. For Irish TIMES, the technologies parameters were all reviewed and revised, as appropriate, for Irish conditions. Each of these technologies also has associated costs (e.g. capital costs, O&M costs, discount rates). In most instances, these costs are input in the form of curves, i.e. as elasticities and as such, they are described as demand curves in that they can meet varying levels of energy demand at varying levels of cost [2].

There are 73 technologies available in the transport sector, including 17 car technologies (see Table 1), 20 bus technologies, 12 road freight truck technologies and 10 train technologies. Fuels options include diesel, gasoline, ethanol, electricity, LPG, natural gas, compressed hydrogen etc.

The outputs from the transport sector include the list of selected technology options in each time period; the associated cost of investment in this suite of technologies; the resulting fuel costs, which are calculated endogenously within the model and are an outputs rather than inputs. The results can distinguish between different fuels used, including the level of electrification, the possible adoption of a number of different types of biofuel and whether these are imported or produced domestically. The model outputs the CO_2_ emissions and can distinguish between direct and indirect emissions. TIMES can also model NOX and SOX emissions.

### 2.3 Drivers

Key data driving the Irish TIMES model are the macro-economic projections of GDP, GNP, private income, population and number of households that is generated using the Economic and Social Research Institute (ESRI) long-term macro-economic model. These parameters are used to generate energy service demand parameters, which are the key quantities that the Irish TIMES model must produce an energy system to satisfy. In total, there are 60 different types of energy services for the transport, residential, agricultural, commercial, industry and non-energy sectors. Some examples include residential space heating (peta-joules, PJ), commercial refrigeration (PJ), industry iron & steel (millions of tonnes, Mt), transport car distance (millions of passenger kilometres, Mpkm) and transport road freight (millions of tonne kilometres, Mtkm). For each modelling period out to 2050, energy service demand parameters are input and the Irish TIMES model must serve these parameters at least cost.

Each energy service demand is projected forward from the base year 2010 to 2050 using exogenously specified demand driver rates and demand elasticities. Demand drivers rates (DDR) and demand elasticities constitute the energy service demand driver (ESD Driver) over the period using the following formulas:

(1) $$DDR(t)=\left(\left(\frac{DemandDriver(t)}{DemandDriver(t-1)}\right)-1\right)$$

(2) $$ESDdriver(t)={\left(1+DDR(t)*elasticity(t)\right)}^{periodlength}*\left(1-AEEI\right)$$

The elasticities were calculated for the period to 2020 by comparing the reference energy scenario within Irish TIMES against Ireland's published national energy forecasts. Table 2 gives the demand driver for each energy service demand and Table 3 provides 5-year projection incremental percentage increases for each of these drivers. Private car transport is driven by gross national product (GNP) with projections taken from the ESRI Medium term review 2013, recovery scenario.

**Table 2:** Transport energy service demands and demand drivers.

Description	Drivers
Car - Long Distance Travel	GNP per Capita
Car - Short Distance Travel	
Motorcycles	GNP
Intercity Bus	Population
Urban Bus	
Passenger Rail - Light	
Passenger Rail - Heavy	
Rail Freight	Transport and Communications GVA
Road Freight	
International Aviation	
Domestic Aviation	
Navigation	
Navigation Bunker

**Table 3:** Transport related driver projections.

Driver	2010–2015	2015–2020	2020–2025	2025–2030	2030–2035	2035–2040	2040–2045	2045–2050
GNP	7%	19%	12%	12%	6%	6%	6%	6%
Population	2%	4%	4%	3%	2%	2%	2%	2%
GNP per Capita	5%	14%	8%	9%	4%	4%	4%	4%
Gross Value Added to Transport and Communications	18%	24%	7%	9%	6%	6%	6%	6%

**Table 4:** Onshore and offshore wind capacities.

Technology	Unit	2006	2010	2015	2020	2025	2030	2050
Onshore Wind	GW	0.3	2.1	3.1	5.3	5.6	5.9	6.9
Offshore Wind	GW	0	0.1	0.6	1	7.7	3.8	7.5

### 2.4 Resource potential and fuels

The resource potential applies mostly to commodities and supply curves, i.e. what is the cost of each commodity at various levels of supply. The resource potential also applies to technologies, particular renewable energy technologies and their resource. There is a limit to the amount of onshore wind power that can be constructed in Ireland based off research from [3], [4], [5], and is summarised in Table 1. The ocean energy resource potential is aligned with the ocean energy roadmap [6] and set at 29 GW in 2050. The maximum capacity for hydro energy has been set at 224 MW for large plants and at 250 MW for run of river plants. The existing 292 MW pumped hydro storage plant is also modelled. The use of geothermal energy in Ireland is limited only to small installations in the residential and services sector mostly for space and water heating purposes. Because solar and geothermal energy contribute marginally to scenarios outputs, no maximum potentials have been provided in the model.

The commodity supply curves and renewable resource for Irish TIMES have been carefully scrutinized and updated based on most recently available data, local knowledge or known technical limits [7].

**Fig. 2:**
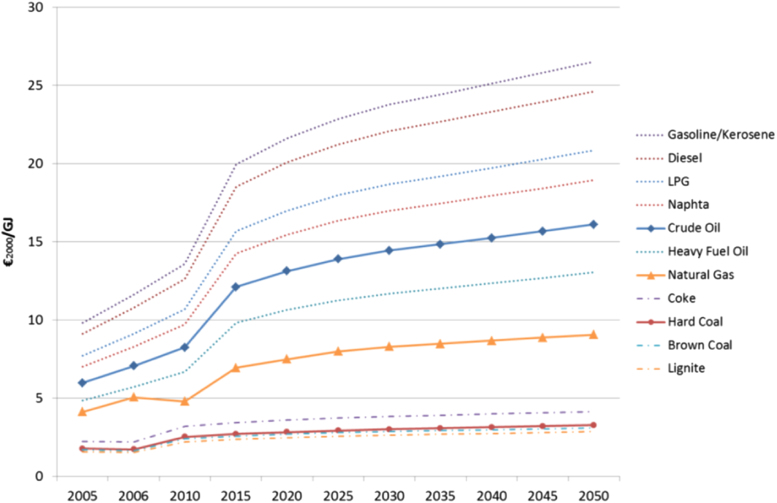
Fuel price projections.

Projections for future fuel prices for key fuel commodities (e.g. coal, oil and gas) are taken from IEA world energy outlook (Fig. 2) [8].

Given the importance of renewable energy for the achievement of mitigation targets, Ireland's energy potentials and costs are based on the most recently available data. The total resource capacity limit for domestic bioenergy has been set at 1,230 ktoe for the year 2020 and at 3,022 ktoe by 2050, based on the estimates listed below (see Table 5).

**Table 5:** Biofuel energy potential.

Commodity Unit	Unit	2005	2010	2020	2030	2040	2050
Agricultural waste^a^	ktoe	25.0	153.1	188.0	188.0	188.0	188.0
Starch crop^a^	ktoe	0.0	31.6	47.4	79.0	79.0	79.0
Grassy crop (Miscanthus)^a^	ktoe	2.7	4.0	28.0	211.3	394.7	910.3
Woody crop (Willow)^a^	ktoe	13.1	19.7	137.6	284.4	431.2	722.0
Forestry residues^a^	ktoe	62.3	93.5	109.1	109.1	109.1	109.1
Biogas^a^^,^^b^	ktoe	30.8	38.4	284.9	382.6	480.3	578.0
Municipal waste^a^	ktoe	71.1	142.2	155.5	155.5	155.5	155.5
Rape seed^b^	ktoe	1.7	7.2	14.3	14.3	14.3	14.3
Industrial waste^a^	ktoe	0.0	2.3	7.0	7.0	7.0	7.0
Wood processing residues^a^	ktoe	258.9	258.9	258.9	258.9	258.9	258.9

a [9].

b [10].

The cost assumptions for domestic bioenergy commodities are based on [11] for biogas from grass, [12] for forestry, [13] for willow and miscanthus crops and [14] for wheat crops. Cost estimates on bioenergy imports are based on an SEAI report by [14] (see Table 6). Cost assumptions for bulk renewable energy technologies were recently updated based on studies by DECC [15] (for wind energy) and [16] (for solar). Electricity prices are calculated endogenously in the model.

**Table 6:** Biofuel energy costs.

Commodity Costs (€2000/GJ)	2005	2010	2020	2030	2040	2050
Agricultural waste	4.10	4.60	5.20	5.20	5.20	5.20
Starch crop	8.16	7.73	7.06	6.59	6.59	6.59
Sugar crop	7.57	7.39	7.15	7.03	7.03	7.03
Grassy crop	4.48	4.30	4.20	4.20	4.20	4.20
Woody crop	2.57	2.41	2.21	2.10	2.10	2.10
Forestry residues	2.74	2.63	2.53	2.53	2.53	2.53
Biogas (from Grass)	4.50	4.10	3.70	3.70	3.70	3.70
Municipal waste	0.80	0.40	0.20	0.20	0.20	0.20
Rape seed	2.74	2.67	2.54	2.43	2.43	2.43
Industrial waste	0.01	0.01	0.01	0.01	0.01	0.01
Wood processing residues	3.25	3.35	3.45	3.45	3.45	3.45

**Table 7:** 2015 Vehicle technology data inputs.

	Mileage (km/yr)	Specific Energy Consumption (MJ/km)
Small Petrol	13,966	1.83
Medium Petrol	17,918	2.22
Large Petrol	18,297	2.70
Small Diesel	17,026	1.60
Medium Diesel	21,370	1.62
Large Diesel	26,088	2.19
Small Hybrid	14,480	1.38
Medium Hybrid	20,110	1.37
Large Hybrid	27,589	1.89
Small Plug in Hybrid	16,372	0.68
Medium Plug in Hybrid	21,628	0.68
Large Plug in Hybrid	22,277	0.77
Battery Electric Vehicle^a^	10,165	0.64

a Mileage for BEVs was taken from the Road Directorate Inspection Data in Denmark, as no mileage on BEVs is currently available for Ireland.

### 2.5 Discount rates

The model uses a general discount rate (year dependent), as well as technology specific discount rates (period dependent). The former is used to: a) discount fixed and variable operating costs, and b) discount investment cost payments from the point of time when the investment actually occurs to the base year chosen for the computation of the present value of the total system cost. The latter are used only to calculate the annual payments resulting from a lump-sum investment in some year. Thus, the only place where the technology specific discount rate intervenes is to compute the Capital Recovery Factors.

Each individual investment physically occurring in year k, results in a stream of annual payments spread over several years in the future. The stream starts in year k and covers years k, k+1, …, k+ELIFE-1, where ELIFE is the economic life of the technology. Each yearly payment is equal to a fraction CRF of the investment cost (CRF = Capital Recovery Factor). Note that if the technology discount rate is equal to the general discount rate, then the stream of ELIFE yearly payments is equivalent to a single payment of the whole investment cost located at year k, in as much as both have the same discounted present value. If however the technology's discount rate is chosen different from the general one, then the stream of payments has a different present value than the lump sum at year k. It is the user's responsibility to choose technology dependent discount rates, and therefore to decide to alter the effective value of investment costs.

In the Irish TIMES economic values are specified in constant Euros of the year 2000. Costs – of building a process, maintenance, or importing a commodity – in year y are given in constant euros of year y, without inflation. Economic values of different years are discounted to the base year 2000 with a general social time preference or real term discount rate. In the Irish TIMES a 6% real term discount rate is assumed, but lower or higher values can be used in sensitivity runs. The technology specific discount rates used in the Irish TIMES for private cars is taken as 17.5%.

### 2.6 CarSTOCK model operation and input assumptions

#### 2.6.1 Technologies

The CarSTOCK model has an extensive list of private car technologies covering both the existing technologies making up the majority of market shares (such as petrol and diesel internal combustion engines (ICES)) along with emerging technologies (hybrids, plug in hybrids (PHEV), and battery electric vehicles (BEV)). Vehicles with a combustion engine, i.e., all vehicles excluding BEVS, are disaggregated by engine size while all vehicles are disaggregated further by vintage.

**Fig. 3:**
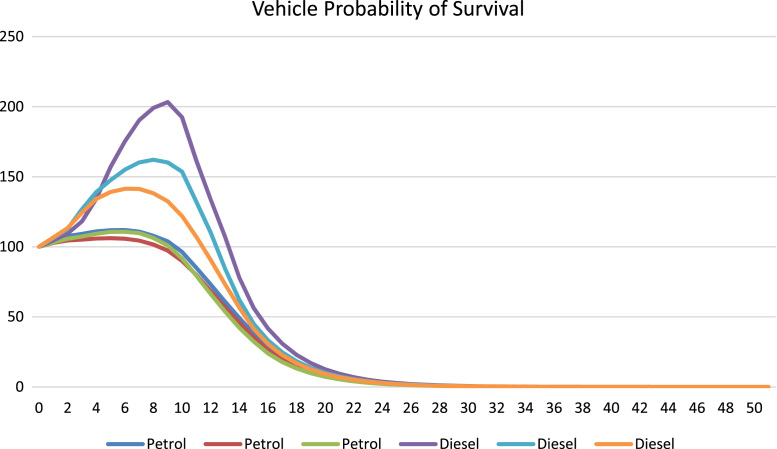
ICE Survival profile.

Base year data was acquired from the Vehicle Registration Unit who provided a detailed list of vehicles by the split detailed above. The survival profile was built for each vehicle of engine size (ES) and vintage (v) using Eq. (3). The resulting probability of survival is presented in Fig. 3.

(3) $$\mathrm{Surviva}{l\;\mathrm{Rate}}_{v}^{\mathrm{ES}}=\mathrm{Average}\left(\frac{\left(\mathrm{Stoc}k_{v}^{\mathrm{ES}}-\mathrm{Stoc}k_{v-1}^{\mathrm{ES}}\right)}{\mathrm{Stoc}k_{v}^{\mathrm{ES}}}\right)*(1+\mathrm{Survival}\;\mathrm{Rat}e_{v-1}^{\mathrm{ES}})$$

Mileage and specific energy consumption of the historic fleet, also disaggregated by engine band, were obtained from the Irish national car test results, a compulsory vehicle inspection in Ireland which records data relating to the road worthiness of all private cars on a bi-annual basis for cars under ten years old, and annually beyond this. Mileage was extrapolated from the average change seen between 2000 and 2015, amounting to a 2% reduction per annum. Specific energy consumption was altered by different rates dependant on the scenario, as outlined by the main manuscript.

### 2.7 Drivers

The drivers of the CarSTOCK model are generated using the Economic and Social Research Institute (ESRI) long-term macro-economic model HERMES results from the Medium term review, 2013. These projections (see Table 3) are linked with income and fuel elasticities of demand from [17] (Table 8).

**Table 8:** Fuel price and income elasticities of demand.

**Elasticities of Demand**	**Stock**	**Vehicle Kilometres**	**Sales**
**Fuel Price Elasticity**	−0.1	−0.1	−0.1
**Income Elasticity**	0.35	0.6	1

These projections linked with the survival profiles and assumptions surrounding mileage and specific energy consumption are used to generate detailed projections of stock, energy, and emissions using the ASIF method (see Eq. (2) of the main manuscript) for the Irish car stock. Finally, market share is calculate exogenously using the market share algorithm outlined below.

### 2.8 Market share algorithm

Heterogeneity is modelled exogenously in the Irish TIMES and CarSTOCK models separately to provide a more realistic market share change based off cost and consumer preference. This method is employed in Irish TIMES by placing a user constraint on the private car sector to represent heterogeneity amongst consumer choice – as the model is based on least cost, a sudden penetration of a cheaper technology void of this added constraint would create a sudden and unrealistic shift in the market share towards this option. This study represents the market uptake of new technologies using the CIMS market share algorithm (see Eq. (4)). CIMS is a hybrid energy-economy model developed at Simon Fraser University that simulates capital stock turnover through time as technologies are acquired, retired, and replaced [18] This equation uses capital costs (CC), maintenance costs (MC), energy costs (EC), intangible costs (i) and a discount rate (r) to calculate the market share of a technology j in year n when competing against K technologies.

(4) $$MS_{j}=\frac{{\left({\mathrm{CC}}_{j}*\frac{r}{1+{\left(1+r\right)}^{-n}}+MC_{j}+{\mathrm{EC}}_{j}+i_{j}\right)}^{-v}}{\sum_{k=1}^{K}{\left({\mathrm{CC}}_{k}*\frac{r}{1+{\left(1+r\right)}^{-n}}+MC_{k}+{\mathrm{EC}}_{k}+i_{k}\right)}^{-v}}$$

This market share algorithm is useful in capturing the effect of the intangible costs associated with alternative fuelled vehicles, such as consumer hesitation towards purchasing new technologies and range anxiety. This intangible cost is calibrated off current market shares in 2013 and 2015, extrapolated to 2050. Capital costs were taken from the current average market prices of vehicles by engine band weighted against the vehicle stock as it stands today. A decrease in the capital cost of pure electric vehicles (PEV) and plug in hybrids (PiH) of 53% over the next 6 years is based on a learning curve assumed from [19]. The fuel costs are taken based off 2015 market prices and projected forward using fuel costs from [20], the discount rate is chosen at 24% and heterogeneity, v, is assumed as 15 based on [21]. This list of parameters is summarised in Table 9. The resulting market shares are entered as a capacity limit for market uptake of private car technologies as a user constraint in Irish TIMES.

**Table 9:** Irish TIMES baseline market share algorithm parameters.

**Technology**	**2015**				**2050**			
	**CC**	**MC**	**EC**	**i**	**CC**	**MC**	**EC**	**i**
Petrol Car	€28,316	€5598	1.26 c/ltr	–	€28,316	€5598	1.66 c/ltr	–
Diesel Car	€28,316	€5598	1.19 c/ltr	–	€28,316	€5598	1.57 c/ltr	–
BEV	€21,490^a^	€5505	0.13 c/kWh	€29,241	€10,041^a^	€5505	0.13 c/kWh	€3843
PHEV	€31,450^b^	€5455	0.81 c/ltr	€10,542	€14,695^b^	€5455	1.05 c/ltr	–

a Price includes government grant of €5000 towards Pure Electric Vehicle purchasing.

b Price includes government grant of €2500 towards Plug in Hybrid Electric Vehicle purchasing.

Similarly, the penetration of alternative fuelled private cars is simulated in the CarSTOCK model through a bounded market share sales to a greater extent, with limitations placed on the maximum penetration over time based on Eq. (4) above. The modelling framework of the CarSTOCK model allows for a greater description of vehicle technologies relative to the TIMES model. Three engine sizes divide petrol and diesel fuelled cars in the model into the engine size classes small (<1300 cc), medium (1301–1900 cc) and large (>1900 cc). Capital costs, operation and maintenance costs, and fuel costs are based off current market prices for all technologies as above, while intangible costs are chosen to account for consumer preference for each technology and are calibrated also using current market shares. Smaller sized vehicles are generally cheaper than their larger sized counterparts, yet larger vehicles tend to have a higher market share, relating to a higher intangible cost due to consumer preference for small vehicles, and a lower intangible cost for larger vehicles (see Table 10).

**Table 10:** CarSTOCK baseline market share algorithm parameters – 2015.

	**CC**	**MC**	**EC**	**i**
**BEV**	€21,490^a^	€5202	0.13 c/kwh	€23,955
**PHEV**	€31,450^b^	€5252	0.81 c/ltr	€15,799
**Petrol - Small**	€14,949	€5261	1.26 c/ltr	€17,362
**Petrol - Medium**	€20,829	€5796	1.26 c/ltr	€14,796
**Petrol - Large**	€43,502	€6666	1.26 c/ltr	€10,008
**Diesel - Small**	€14,995	€5261	1.19 c/ltr	€25,987
**Diesel - Medium**	€24,180	€5796	1.19 c/ltr	€9770
**Diesel - Large**	€43,705	€6666	1.19 c/ltr	€78

a Price includes government grant of €5000 towards Pure Electric Vehicle purchasing.

b Price includes government grant of €2500 towards Plug in Hybrid Electric Vehicle purchasing.
