# Supplementary material for: Techno-economic data for a multi-model approach to decarbonisation of the Irish private car sector
Source: Data Brief. 2017 Oct 7;15:922–32. doi: 10.1016/j.dib.2017.10.006 (PMC5683748; doi:10.1016/j.dib.2017.10.006)
Supplement: Supplementary file 1 — Supplementary material [file mmc1.docx]

CONFLICT OF INTEREST FORM

We wish to confirm that there are no known conflicts of interest

associated with this publication and there has been no significant financial support for this work that could have influenced its outcome.

We confirm that the manuscript has been read and approved by all named authors and that there are no other persons who satisfied the criteria for authorship but are not listed. We further confirm that the order of authors listed in the manuscript has been approved by all of us.

We confirm that we have given due consideration to the protection of intellectual property associated with this work and that there are no impediments to publication, including the timing of publication, with respect to intellectual property. In so doing we confirm that we

have followed the regulations of our institutions concerning intellectual property.

Signed by all authors as follows:

**
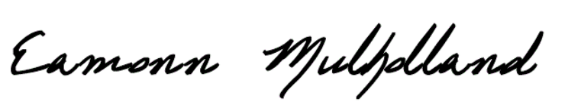
**

Eamonn Mulholland


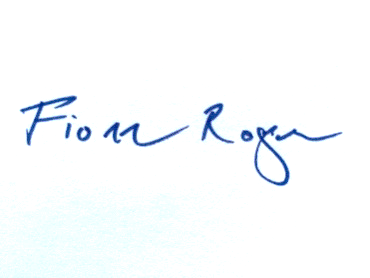


Fionn Rogan


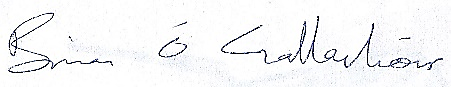


Brian Ó Gallachóir
